# Naturally sterile *Mus spretus* hybrids are suitable for the generation of pseudopregnant embryo transfer recipients

**DOI:** 10.1038/s41684-024-01393-4

**Published:** 2024-06-17

**Authors:** Chris Preece, Daniel Biggs, Edward Grencis, Maj Simonsen Jackson, Sue Allen, Martin Fray, Antony Adamson, Benjamin Davies

**Affiliations:** 1 Wellcome Centre for Human Genetics, Oxford, UK; 2 University of Manchester, Manchester, UK; 3 Mary Lyon Centre at MRC Harwell, Didcot, UK; 4 The Francis Crick Institute, London, UK

**Keywords:** Animal breeding, Genetic engineering

## Abstract

For the preparation of embryo transfer recipients, surgically vasectomized mice are commonly used, generated by procedures associated with pain and discomfort. Sterile transgenic strains provide a nonsurgical replacement, but their maintenance requires breeding and genotyping procedures. We have previously reported the use of naturally sterile STUSB6F1 hybrids for the production of embryo transfer recipients and found the behavior of these recipients to be indistinguishable from those generated by vasectomized males. The method provides two substantial 3R impacts: refinement (when compared with surgical vasectomy) and reduction in breeding procedures (compared with sterile transgenic lines). Despite initial promise, the 3Rs impact of this innovation was limited by difficulties in breeding the parental STUS/Fore strain, which precluded the wider distribution of the sterile hybrid. The value of a 3R initiative is only as good as the uptake in the community. Here we, thus, select a different naturally sterile hybrid, generated from strains that are widely available: the B6SPRTF1 hybrid between C57BL/6J and *Mus spretus*. We first confirmed its sterility by sperm counting and testes weight and then trialed the recovery of cryopreserved embryos and germplasm within three UK facilities. Distribution of sperm for the generation of these hybrids by in vitro fertilization was found to be the most robust distribution method and avoided the need to maintain a live *M. spretus* colony. We then tested the suitability of B6SPRTF1 sterile hybrids for the generation of embryo transfer recipients at these same three UK facilities and found the hybrids to be suitable when compared with surgical vasectomized mice and a sterile transgenic strain. In conclusion, the potential 3Rs impact of this method was confirmed by the ease of distribution and the utility of sterile B6SPRTF1 hybrids at independent production facilities.

## Abstract (web summary)

Preece et al. show that B6SPRTF1 hybrid males between C57BL/6J and *Mus spretus* are suitable for the generation of pseudopregnant female mice for embryo transfer. By providing an alternative to vasectomized males, the method shows clear 3R benefits.

## Main

The generation, rederivation and distribution of genetically altered mice strains necessitates the production of pseudopregnant mice that serve as recipients for embryos^1^. Before transfer, the transferred embryos have either been manipulated genetically via microinjection or electroporation of DNA constructs or site-specific nucleases or simply transported to a facility as either fresh or frozen embryos.

Traditionally, surgical vasectomy was routinely used in animal facilities to prepare male mice that could serve as studs for the generation of pseudopregnant embryo transfer recipients^2^. More recently, in an initiative to refine and replace procedures of moderate severity, many facilities now rely upon genetically altered mice strains, which manifest a male infertility phenotype. One particular strain, *Tg(Prm1-eGFP)#Ltku*, is frequently used and is particularly practical because the transgenic status of the sterile male mice is indicated by green fluorescence, facilitating the genotyping and identification of putative sterile males^3^. Other genetically modified strains have also been used for this concept of a genetic vasectomy; for example, male *Gapdhs* knockouts are sterile^4^.

Although reliance on these genetically altered strains avoids the need for surgical procedures, their use necessitates the maintenance of a colony of genetically altered mice. The transgenic lines showing male sterility must be bred from female hemizygous mice, and genetically sterile knockout mice must be bred by intercrossing heterozygous mice. Accordingly, using genetically altered sterile strains necessitates a large breeding initiative and accompanying genotyping procedures.

As an alternative, we have previously reported the use of naturally sterile hybrids of two subspecies—an F1 cross between the *Mus musculus musculus* substrain (STUS/Fore) and the *Mus musculus domesticus* strain (C57BL/6J)—for the preparation of pseudopregnant embryo transfer recipients^5^. The behavior of embryo transfer recipients generated using these STUSB6F1 mice was found to be indistinguishable from those generated by vasectomized males. Furthermore, when compared with the use of genetically sterile mice, the generation of these hybrids is more efficient (all male mice produced from the intercross are sterile), and the breeding involves only wild-type mice and is, thus, not regulated under animal procedure legislation.

Despite the 3Rs impact of the use of the STUSB6F1, the method necessitates the maintenance of a STUS/Fore colony^6^. It was found that the maintenance of this strain was inefficient due to a small litter size and frequent lack of fecundity in the mice. Resourcing the strain from its origin in research institutes in Czechia was unsuccessful. Subsequently, given that we were unable to disseminate the method to other facilities, we investigated the use of alternative sterile hybrids that can be generated from more common strains.

In this Article, we select the naturally sterile male offspring which result from an interspecies intercross between male *Mus spretus* and female *M. musculus* (C57BL/6J) mice^7–9^. In contrast with the STUS/Fore model, *M. spretus* is available from international repositories and is more widespread in academic facilities. With an aim of maximizing the 3Rs impact of this strain, we tested their use for the production of pseudopregnant embryo transfer recipients at three different UK biomedical facilities and compared their performance against both surgical vasectomized mice and the genetically sterile *Tg(Prm1-eGFP)#Ltku* mice^3^ (Supplementary Fig. 1). The results at all three centers confirmed the suitability of the B6SPRTF1 hybrid males for embryo transfer and allowed an optimization of the distribution of this strain.

## Results

**Fig. 1:**
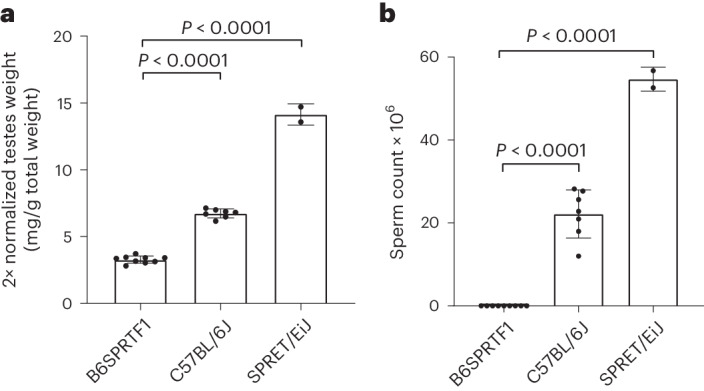
Confirmation of the infertility of B6SPRTF1 male hybrid mice. **a**, Normalized testes weight for B6SPRTF1 (*n* = 9) as compared with the two parental strains, C57BL/6J (*n* = 7) and SPRET/EiJ (*n* = 2). **b**, As **a** but showing the total caudal sperm count (two-sided Student’s *t*-test; *P* < 0.0001 for all comparisons). Source data

Male offspring of a cross between male *M. spretus* (Spret/EiJ) and female *M. musculus* (C57BL/6J) are sterile due to a failure of chromosome synapsis during the meiotic prophase^9,10^. We first confirmed the fertility phenotype in these hybrids, finding both significantly reduced testes size when compared with the parental wild-type strains and a complete lack of sperm in the hybrids (Fig. 1).

The aim of our study is not only to select a naturally sterile hybrid strain but also to ensure that its distribution to other facilities was sufficiently robust, maximizing the 3Rs benefits of its potential use. We, thus, generated large numbers of B6SPRTF1 embryos at the University of Oxford (UoO) and cryopreserved them at the two-cell stage for distribution. The embryos were shipped to two UK facilities (Mary Lyon Centre (MLC) and the University of Manchester (UoM)) for thawing and rederivation; thawing and rederivation also occurred within the UoO facility. At all three sites, a relatively poor recovery of live births was obtained following the thawing and rederivation of the cryopreserved B6SPRTF1 two-cell embryos (Table 1). This result suggests that the cryopreservation or thawing parameters (media, conditions, and so on) would need to be optimized to ensure efficient embryo recovery.

**Table 1:** Rederivation efficiency using frozen and fresh B6SPRTF1 embryos

Facility	Frozen embryos			Fresh embryos prepared from IVF		
	Embryos transferred (*N*)	Pups born (*N*)	Birth rate (%)	Embryos transferred (*N*)	Pups born (*N*)	Birth rate (%)
UoO	220	40	18.8	98	33	33.7**
MLC	127	9	7.1	135	20	14.8*
UoM	131	14	10.6	–	–	–

**Chi square of 8.4; d.f. of 1; *P* = 0.004. *Chi square of 3.9; d.f. of 1; *P* = 0.048.

**Fig. 2:**
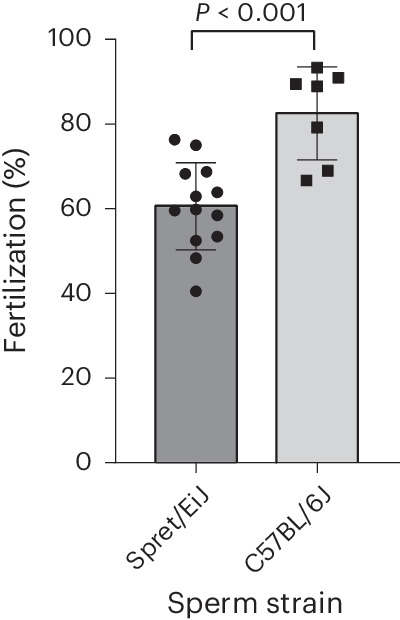
IVF rates. Comparison of fertilization rates (number of two-cell embryos following overnight culture as a fraction of total oocytes used for IVF) of independent sessions using C57BL/6J oocytes with either *M. spretus* (Spret/EiJ) (*n* = 13) or C57BL/6J (*n* = 7) thawed sperm (chi-squared test; *P* < 0.001). Source data

As an alternative, we explored the possibility of distributing cryopreserved sperm from the Spret/EiJ strain for production and transfer of live B6SPRTF1 embryos. We first began by cryopreserving Spret/EiJ sperm from live mice at the UoO and then verified the use of in vitro fertilization (IVF) to generate B6SPRTF1 using thawed sperm from Spret/EiJ mice and oocytes prepared from superovulated C57BL/6J females. Over a number of independent sessions, a good rate of IVF (mean 60.6%), as defined by two-cell embryo progression following overnight culture, was observed, albeit at a lower rate than that obtained with thawed C57BL/6J sperm (mean 82.5%) (Fig. 2). Cryopreserved Spret/EiJ sperm, prepared at the UoO, was then shipped to the MLC facility to allow IVF and rederivation with freshly generated embryos to be tested. Unlike frozen and thawed B6SPRTF1 embryos, the transfer of these live two-cell embryos at both the UoO and MLC facilities led to a significant increase in the efficiency of live pup production (Table 1; UoO, *P* < 0.001; MLC, *P* < 0.05). This method was, thus, selected as the method of choice for the distribution of this strain.

Having established the best distribution methodology, the three sites explored the suitability of the sterile B6SPRTF1 hybrid strains for the generation of pseudopregnant embryo transfer recipients. UoO prepared recipients for both oviduct transfer of genetically manipulated two-cell embryos and uterine transfer of blastocysts, comparing the results with historical data from surgically vasectomized males. The UoM used the embryo transfer recipients for oviduct transfer of genetically manipulated and unmanipulated (rederivation) two-cell embryos and compared the results with contemporaneous embryo transfer recipients prepared from vasectomized CD1s. The MLC used the embryo transfer recipients for oviduct transfer of nonmanipulated embryos (rederivation) and compared the results with contemporaneous embryo transfer recipients prepared using genetically sterile *Tg(Prm1-eGFP)#Ltku*^3^.

**Fig. 3:**
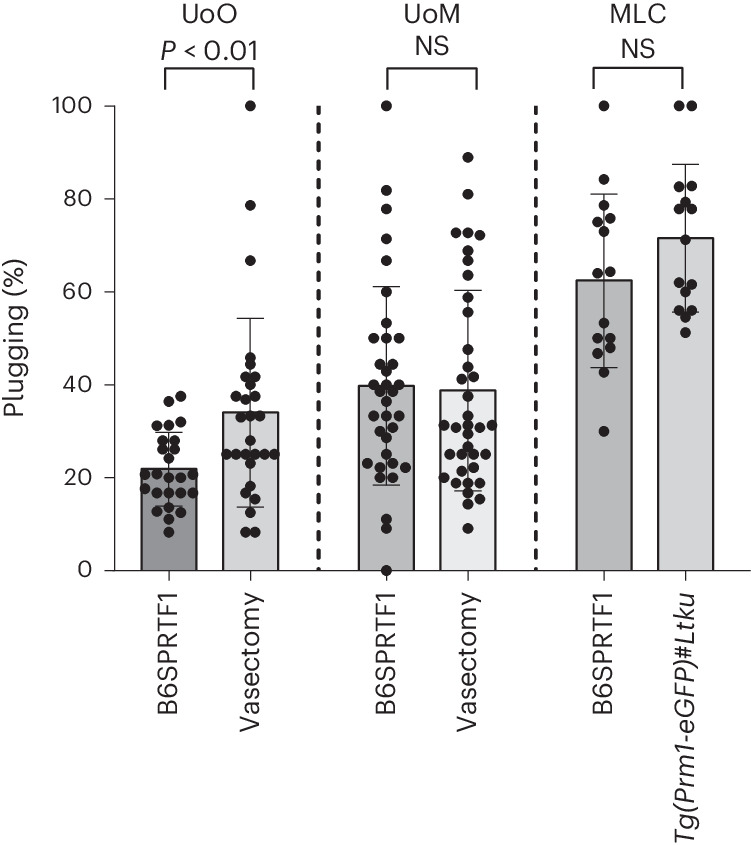
Plugging efficiencies of sterile mice. Plugging rates observed per week (percentage of females plugged by sterile males) at each of the three facilities, comparing the B6SPRTF1 studs (UoO, *n* = 25 (data collected November 2021–August 2022); UoM, *n* = 35; MLC, *n* = 15) with either vasectomized mice (UoO, *n* = 28 (data collected January 2018–January 2019); UoM, *n* = 37) or genetically sterile *Tg(Prm1-eGFP)#Ltku* mice (MLC, *n* = 15) (chi-squared test, *P* > 0.05 for UoM and MLC and *P* < 0.01 for UoO). NS, not significant. Source data

**Extended Data Fig. 1:**
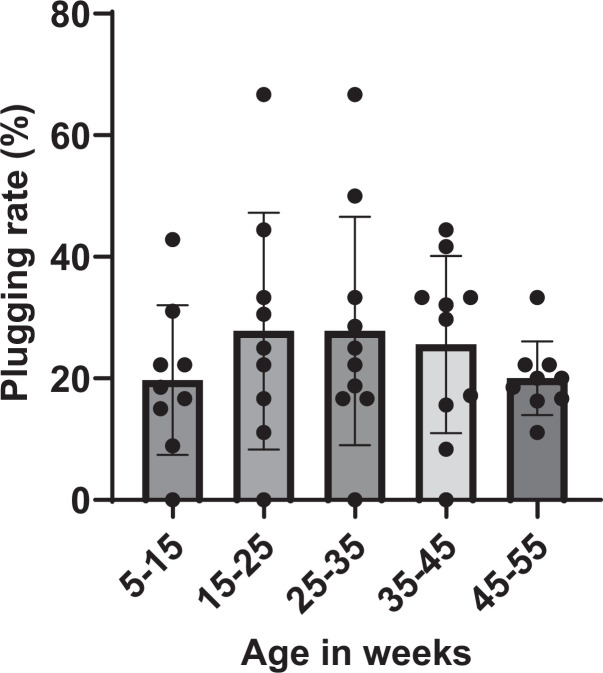
Effect of age on B6SPRTF1 plugging efficiency. Plugging rates observed per week (percentage of females plugged by sterile males) is shown for B6SPRTF1 at various ages. There was no significant effect of age on performance observed. Source data

We first compared the plugging rate of CD1 females obtained with B6SPRTF1 sterile hybrid males to that obtained with vasectomized or genetically sterile males and observed varying results (Fig. 3). In the UoO facility, a significant reduction in plugging efficiency was observed for the sterile hybrids compared with surgically vasectomized studs. However, this difference, which might reflect the fact that the comparison was performed using historical data, could not be replicated at the UoM facility which reported no difference in plugging behavior between these same two stud groups when the comparison was performed contemporaneously. The MLC facility also reported no significant differences in plugging efficiency between the B6SPRTF1 sterile hybrids when compared with the genetically sterile *Tg(Prm1-eGFP)#Ltku*. A longer-term study at the UoO revealed that the plugging efficiency was stable and did not decline with age (Extended Data Fig. 1), in line with what is known about the reproductive longevity of vasectomized mice.

Following embryo transfer into a pseudopregnant CD1 female, there was no significant difference in pregnancy rate, as defined by the percentage of surgically transferred females becoming pregnant, between the foster mothers that had been prepared with B6SPRTF1 sterile hybrid males or the comparison group (vasectomized or *Tg(Prm1-eGFP)#Ltku*) for both blastocyst transfer into the uterus (UoO) or two-cell embryo transfer into the oviduct at all three facility sites (Table 2).

**Table 2:** Pregnancy rates, as defined by the percentage of females becoming pregnant following surgical transfer into pseudopregnant foster mothers prepared via either sterile B6SPRTF1, surgically vasectomized or genetically sterile studs

Facility	Transfer type	Pregnancy rate			Between group comparison
		B6SPRTF1	Vasectomy	*Tg(Prm1-eGFP)#Ltku*	
UoO	Uterine	75.0% (*n* = 44)	69.2% (*n* = 107)	–	Chi square of 0.54; *P* > 0.05
UoO	Oviduct	75.5% (*n* = 184)	83.1% (*n* = 154)	–	Chi square of 2.91; *P* > 0.05
MLC	Oviduct	63.2% (*n* = 299)	–	67.1% (*n* = 603)	Chi square of 1.35; *P* > 0.05
UoM	Oviduct	53.9% (*n* = 115)	68.2% (*n* = 44)	–	Chi square of 2.65; *P* > 0.05

**Fig. 4:**
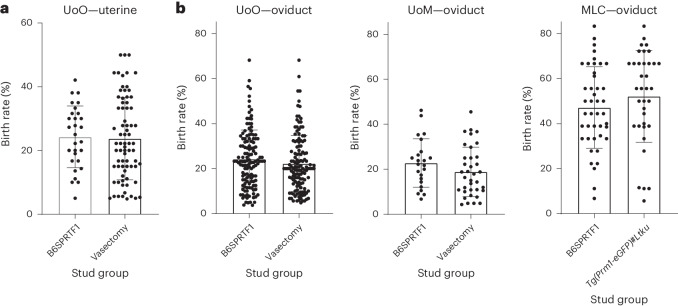
Embryo transfer birth rates. **a**,**b**, Birth rates (the number of live born as a percentage of the number of embryos transferred) from each transferred pseudopregnant mouse, prepared with either B6SPRTF1 males, surgically vasectomized males or genetically sterile *Tg(Prm1-eGFP)#Ltku* males. The birth rates (in percent) are shown for uterine transfer of blastocysts at UoO (**a**) and oviduct transfer of gene-edited embryos at UoO and UoM or nonmanipulated embryos at MLC (**b**) (chi-squared test, *P* > 0.05 for all pairwise comparisons). Source data

With respect to the birth rate following embryo transfer, defined by the number of live born as a percentage of the number of embryos transferred, all facilities observed no significant differences (chi-squared test, *P* > 0.05 for all pairwise comparisons) between pseudopregnant embryo transfer recipients prepared using either naturally sterile B6SPRTF1, surgically vasectomized mice or genetically sterile *Tg(Prm1-eGFP)#Ltku* studs for both uterine transfer of blastocysts (UoO; Fig. 4a) or oviduct transfer of two-cell embryos (UoO, UoM and MLC; Fig. 4b). The birth rate at the MLC was higher than in the other facilities, which is explained by the fact that this facility performed embryo transfer only with unmanipulated embryos, in comparison with the UoO and UoM facilities, which used microinjected or electroporated embryos.

**Fig. 5:**
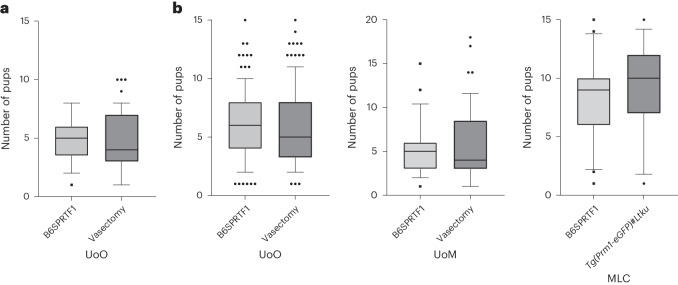
Average litter size. **a**,**b**, Box plot showing the range (10th–90th percentile) of litter sizes obtained from each transferred pseudopregnant mouse, prepared with either B6SPRTF1 males, surgically vasectomized males or genetically sterile *Tg(Prm1-eGFP)#Ltku* males. The litter sizes for uterine transfer of blastocysts at UoO (**a**) and oviduct transfer of gene-edited embryos at UoO and UoM or nonmanipulated embryos at MLC are shown (**b**) (Mann–Whitney test; *P* > 0.05 for all pairwise comparisons). Source data

No significant differences were observed in the average litter size born from successfully implanted and pregnant foster mothers prepared by mating with the sterile B6SPRTF1 studs or the comparison group (surgically vasectomized mice or genetically sterile *Tg(Prm1-eGFP)#Ltku*) (Fig. 5), with each facility transferring a matched number of embryos across the two experimental groups.

## Discussion

Across three independent facilities, we have demonstrated that the source of the sterile studs used to prepare pseudopregnant embryo transfer recipients has no impact on the production statistics following embryo transfer. Pregnancy rate, birth rate and average litter size were all not significantly affected by how the pseudopregnant female was prepared.

The only significant difference was the observation, made in the UoO facility, that the B6SPRTF1 studs plugged less efficiently than surgically vasectomized mice. This difference could be due to the fact that the comparison of the B6SPRTF1 plugging efficiency within this facility was performed with historical data collected from surgically vasectomized males several years prior. Subsequently, it cannot be ruled out that environmental or personnel factors (for example, a different technician recording the plugs) may have contributed to this difference. Another contributing factor could be the size difference between the B6SPRTF1 males and the CD1 females, as the B6SPRTF1 males, when used at 6 weeks of age, were found to be substantially smaller than an age-matched CD1 mouse. However, in the UoO data, there was no obvious improvement in plugging efficiency as the B6SPRTF1 male mouse matured (Extended Data Fig. 1). Intriguingly, this difference in plugging efficiency was not observed when the same comparison was performed in an independent facility (UoM) and, thus, the historical comparison is the most probable explanation for the plugging difference observed at this one facility.

Overall, the difference in plugging efficiency between facilities is striking, but this difference reflects the different ways in which the plugged females were generated. At the UoO, the females were distributed to the stud cages without any prior exposure to the males for the individual night or nights preceding the mating. In contrast at the UoM, females were continually mated with studs, the exposure thus providing a priming for estrous (Whitten effect^11^) that might explain a higher plugging efficiency. At the MLC, the Whitten effect was used systematically with all females being primed by direct contact with male mice before mating, which explains the increased and more reliable plugging rate observed within this facility.

Overall, despite this potential difference in plugging efficiency, the use of the B6SPRTF1 hybrids provides two substantial 3R impacts. When compared with surgical vasectomy, the method is a clear: refinement (replacing the procedure of surgical vasectomy) and a reduction in breeding procedures (when compared with the use of sterile genetically altered lines). By contrast with the breeding of genetically modified strains with a male sterility phenotype, all male mice produced from an intersubspecific cross are sterile.

The 3Rs impact of a method improvement is only as good as its uptake in the community. Our previous study showing the suitability of the STUSB6F1 males for the preparation of pseudopregnant females led to a large number of requests for these hybrids from mouse facilities. However, due to the difficulties in maintaining the parental STUS/Fore line, it quickly became apparent that dissemination of this potential 3Rs initiative was challenging^5^.

The use of *M. spretus* hybrids as an alternative overcomes these issues for several reasons. First, *M. spretus* is freely available in numerous academic facilities and at several key international repositories (The Jackson Laboratory Strain no. 001146 Strain, Riken Strain RBRC00208). Second, the husbandry required for the preparation of the B6SPRTF1 sterile hybrid is easier because *M. spretus* males are used with C57BL/6J females; by contrast, the STUSB6F1 hybrid required the use of STUS/Fore females with C57BL/6J males (the reciprocal cross was found to be only partially sterile^5^). Third, the direction of the cross using *M. spretus* males allows for the hybrids to be generated by IVF with archived *M. spretus* sperm^7^, obviating the need for a live *M. spretus* breeding colony in facilities wishing to implement this approach.

Despite the convenience of generating the sterile hybrids via IVF, one must consider that this production approach would require additional animal procedures, namely the hormonal stimulation of the oocyte donors (typically two intraperitoneal injections), and the resulting surgical embryo transfer procedures. It is also important to note that the IVF rate when using *M. spretus* sperm was found to be significantly lower than seen when using C57BL/6J sperm, potentially because the Infrafrontier protocols used are optimized for the C57BL/6J strain. Potentially, new protocol improvements in assisted reproductive techniques optimized for *M. spretus* may help improve these rates^12^.

In a small facility with only a few dozen sterile males in use, despite the lower fertilization rate, we would argue that the welfare impact of these procedures is offset by the 3Rs benefit of not having to perform surgical vasectomy on all male mice or to maintain a colony of genetically altered sterile mice. This is simply because there is a good probability that multiple male sterile mice would be born from a single surgical embryo transfer (a clear reduction than having to perform surgical procedures on each wild-type male). For larger facilities with requirements for greater numbers of sterile males and for facilities who would aim to maximize the 3Rs impact of this approach, it would be recommended to generate the B6SPRTF1 hybrids by natural matings of a *M. spretus* male with a C57BL/6J female. This wild-type mating would not be classified as an animal procedure under most legislation and has no significant welfare concerns (see ref. ^13^ for further discussion on strategies) This production approach would clearly necessitate a live *M. spretus* colony to be maintained, to ensure an, albeit low level, supply of male studs for the production of sterile hybrids. Alternatively, live male mice could be purchased intermittently from international repositories when production of sterile hybrids is required. Maintaining a colony and/or purchasing from the repositories also comes at a financial cost which, although perhaps not relevant to the discussions on 3Rs impact, has important logistical and/or feasibility considerations.

Our validation of this hybrid at three independent facilities confirms the suitability and reproducibility of the approach; it will also aid the replacement of either surgically vasectomized males or genetically sterile males for the preparation of embryo transfer recipients widely.

## Methods

### Animal experiments

Wild-type *M. spretus* mice were obtained from the Jackson laboratories (strain no. 001146) and wild-type C57BL/6J and CD1 mice were obtained from Charles River Laboratories (UoO and UoM) or bred in house (MLC). *Tg(Prm1-eGFP)#Ltku* were obtained from Pawel Pelczar, University of Basel. In contrast to the parental strain, *M. spretus*, the B6SPRTF1 were robust and needed no special husbandry conditions. The mice were housed in individually ventilated cages in a 12:12 h light cycle, with access to food and water ad libitum. All studies were conducted with ethical approval by the institutional Animal Welfare and Ethical Review Body and in accordance with the UK Home Office Animals (Scientific Procedures) Act 1986 (UoO: PPL PAA2AAE49, UoM: PPL PP3720525/PP9997444, MLC: PPL PP9563804). Because the difference in color of the mice (albino CD1 versus agouti B6SPRTF1), the experiments could not be carried out with blinding. All three facilities were held between 20 and 24 °C and at 55 ± 10% humidity.

### IVF and cryopreservation

These techniques were performed using the Infrafrontier protocols (https://infrafrontier.eu/emma/cryopreservation-protocols/). Sperm was cryopreserved using CPA supplemented with l-glutamine and ten straws of sperm were collected from each *M. spretus* mouse. IVF was performed using a single straw of thawed *M. spretus* sperm together with oocytes from three superovulated C57BL/6J females. The fertility rate was calculated as the number of two-cell embryos obtained following overnight culture as a fraction of the total oocytes used for IVF.

### Sterility assessment

Males were culled at 10 weeks of age, and sperm counts were obtained by dissection of the caudal epididymis and dispersal of the sperm in 1,000 μl of warm phosphate-buffered saline before counting with a hemocytometer. The paired testes weight was normalized to total body weight.

### Surgical vasectomy

Male CD1 mice at 5 weeks of age were surgically vasectomized by using the scrotal incision technique^2^ and left to recover for ≥3 weeks before use. All vasectomized mice were test bred with 8-week-old CD1 females to ensure sterility before use in embryo transfer experiments.

### Plugging of pseudopregnant embryo transfer recipient

A plugging rate was calculated as the percentage of females plugged by a sterile male per week. Substantial procedural differences were present between facilities. For the UoO, female mice were not routinely exposed to males before mating. For the UoM, female mice were continually mated until the plugs were obtained and the females removed. For the MLC, female mice were systematically exposed to male contact 3 days before the mating. The plugging was assessed for approximately 24 weeks at the MLC, 37 weeks at the UoM and 52 weeks at the UoO.

### Surgical embryo transfer

Mature CD1 (8–16-week-old) females were mated with either vasectomized CD1 males, sterile B6SPRTF1 males (10–30 weeks of age) or transgenic *Tg(Prm1-eGFP)#Ltku*^3^ mice to induce pseudopregnancy, and successful mating was confirmed by the presence of a vaginal plug the following morning. Oviduct transfer of mouse two-cell embryos (average number of embryos transferred: UoO, 19.4 ± 2.4; UoM, 19.4 ± 2.9; MLC, 18.1 ± 2.0) or uterine transfer of mouse blastocysts (UoO: average number of blastocysts transferred: 26.2 ± 5.3) were performed as described^1^. The pregnancy rate was defined as the percentage of surgically transferred females becoming pregnant. The birth rate was defined as the number of live born as a percentage of the number of embryos transferred.

### Statistical analysis

All measurements were taken from distinct samples. The data sets were analyzed and presented by using Graphpad Prism software v8.4 and measurements from distinct samples. The differences between groups were assessed by using a two-tailed Student’s *t*-test (for testes weight and sperm count), chi-squared test for comparison of proportions (for plugging, pregnancy and birth rates) and Mann–Whitney tests for analysis of litter size.

### Reporting summary

Further information on research design is available in the Nature Portfolio Reporting Summary linked to this article.

## Online content

Any methods, additional references, Nature Portfolio reporting summaries, source data, extended data, supplementary information, acknowledgements, peer review information; details of author contributions and competing interests; and statements of data and code availability are available at 10.1038/s41684-024-01393-4.

### Supplementary information

Supplementary information Supplementary Fig. 1 Schematic of the study design.

Reporting Summary

### Source data

Source Data Fig. 1

Source Data Fig. 2

Source Data Fig. 3

Source Data Fig. 4 and Source Data Fig. 5

Source Data Extended Data Fig.1 and Table 1

## Data Availability

All data supporting the findings of this study are available within the paper and its supplementary information. Source data are provided with this paper.
