# Supplementary material for: Naturally sterile Mus spretus hybrids are suitable for the generation of pseudopregnant embryo transfer recipients
Source: Lab Anim (NY). 2024 Jun 17;53(7):181–5. doi: 10.1038/s41684-024-01393-4 (PMC11216974; doi:10.1038/s41684-024-01393-4)
Supplement: Supplementary file 1 — Supplementary Fig. 1 Schematic of the study design. [file 41684_2024_1393_MOESM1_ESM.pdf]

lab animal

https://doi.org/10.1038/s41684-024-01393-4

Naturally sterile *Mus spretus* hybrids are suitable for the generation of pseudopregnant embryo transfer recipients

In the format provided by the authors and unedited

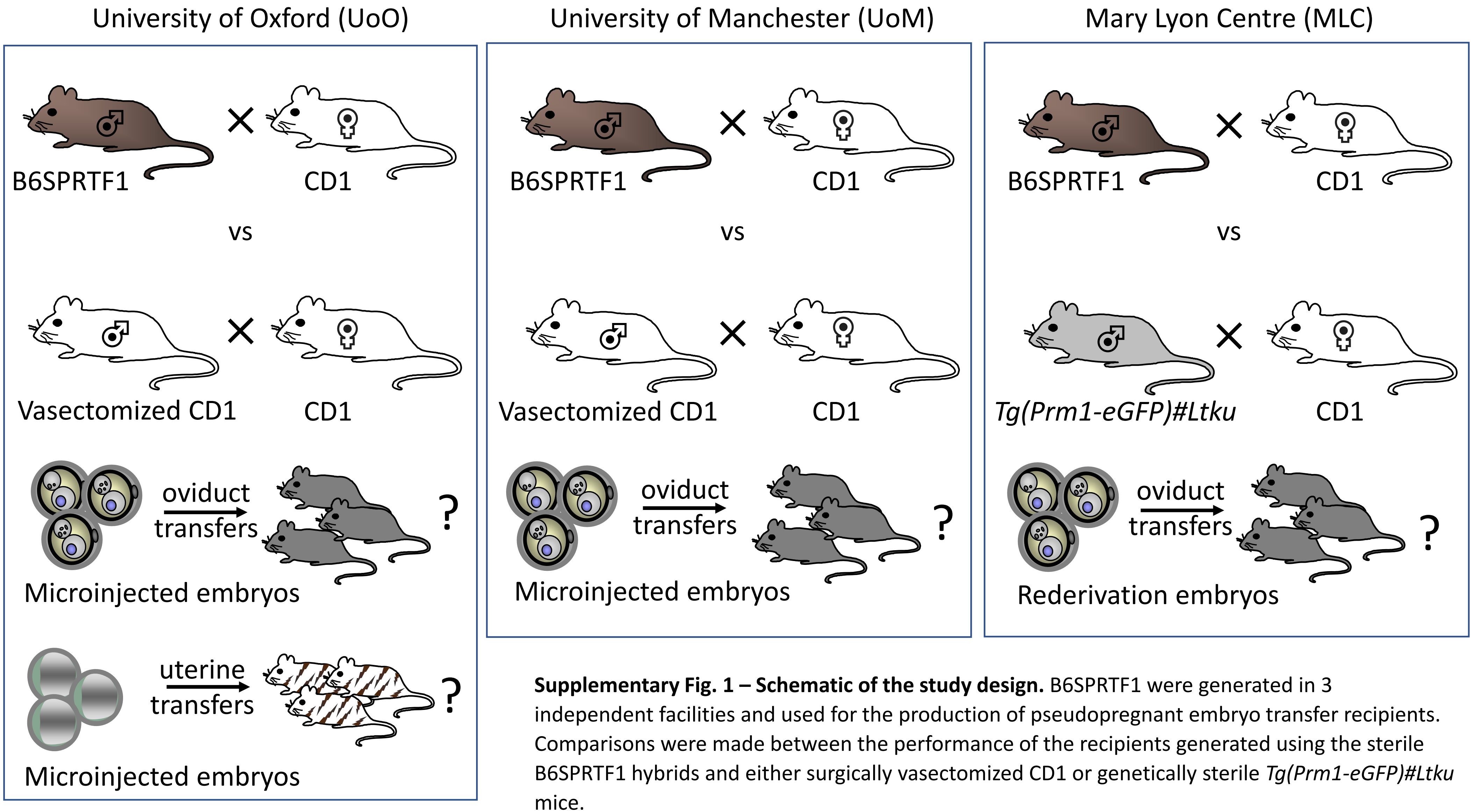

Supplementary Fig. 1 – Schematic of the study design. B6SPRTF1 were generated in 3 independent facilities and used for the production of pseudopregnant embryo transfer recipients. Comparisons were made between the performance of the recipients generated using the sterile B6SPRTF1 hybrids and either surgically vasectomized CD1 or genetically sterile *Tg(Prm1-eGFP)#Ltku* mice.
