# Supplementary material for: Naturally sterile Mus spretus hybrids are suitable for the generation of pseudopregnant embryo transfer recipients
Source: Lab Anim (NY). 2024 Jun 17;53(7):181–5. doi: 10.1038/s41684-024-01393-4 (PMC11216974; doi:10.1038/s41684-024-01393-4)
Supplement: Supplementary file 2 — Reporting Summary [file 41684_2024_1393_MOESM2_ESM.pdf]

nature portfolio

Corresponding author(s): Benjamin Davies

Last updated by author(s): Sep 22, 2023

Reporting Summary

Nature Portfolio wishes to improve the reproducibility of the work that we publish. This form provides structure for consistency and transparency in reporting. For further information on Nature Portfolio policies, see our Editorial Policies and the Editorial Policy Checklist.

Statistics

For all statistical analyses, confirm that the following items are present in the figure legend, table legend, main text, or Methods section.

n/a	Confirmed	
☐	☒	The exact sample size (*n*) for each experimental group/condition, given as a discrete number and unit of measurement
☐	☒	A statement on whether measurements were taken from distinct samples or whether the same sample was measured repeatedly
☐	☒	The statistical test(s) used AND whether they are one- or two-sided<br>*Only common tests should be described solely by name; describe more complex techniques in the Methods section.*
☐	☒	A description of all covariates tested
☒	☐	A description of any assumptions or corrections, such as tests of normality and adjustment for multiple comparisons
☒	☐	A full description of the statistical parameters including central tendency (e.g. means) or other basic estimates (e.g. regression coefficient) AND variation (e.g. standard deviation) or associated estimates of uncertainty (e.g. confidence intervals)
☐	☒	For null hypothesis testing, the test statistic (e.g. *F*, *t*, *r*) with confidence intervals, effect sizes, degrees of freedom and *P* value noted<br>*Give P values as exact values whenever suitable.*
☒	☐	For Bayesian analysis, information on the choice of priors and Markov chain Monte Carlo settings
☒	☐	For hierarchical and complex designs, identification of the appropriate level for tests and full reporting of outcomes
☒	☐	Estimates of effect sizes (e.g. Cohen's *d*, Pearson's *r*), indicating how they were calculated

Our web collection on statistics for biologists contains articles on many of the points above.

Software and code

Policy information about availability of computer code

Data collection	n/a
Data analysis	Data was analyzed using Graphpad Prism v8.4

For manuscripts utilizing custom algorithms or software that are central to the research but not yet described in published literature, software must be made available to editors and reviewers. We strongly encourage code deposition in a community repository (e.g. GitHub). See the Nature Portfolio guidelines for submitting code & software for further information.

Data

Policy information about availability of data

All manuscripts must include a data availability statement. This statement should provide the following information, where applicable:

- Accession codes, unique identifiers, or web links for publicly available datasets
- A description of any restrictions on data availability
- For clinical datasets or third party data, please ensure that the statement adheres to our policy

All data supporting the findings of this study are available within the paper and its Supplementary Information.

Human research participants

Policy information about studies involving human research participants and Sex and Gender in Research.

Reporting on sex and gender	n/a
Population characteristics	n/a
Recruitment	n/a
Ethics oversight	n/a

Note that full information on the approval of the study protocol must also be provided in the manuscript.

Field-specific reporting

Please select the one below that is the best fit for your research. If you are not sure, read the appropriate sections before making your selection.

☒ Life sciences ☐ Behavioural & social sciences ☐ Ecological, evolutionary & environmental sciences

For a reference copy of the document with all sections, see nature.com/documents/nr-reporting-summary-flat.pdf

Life sciences study design

All studies must disclose on these points even when the disclosure is negative.

Sample size	Sample size was determined by a power calculation assuming a dichotomous endpoint (pregnant/non-pregnant) and assuming that a 25% difference between the groups in pregnancy rate would be unacceptable, and with anability of detect this decrease with 80% power and at 0.05% significance.
Data exclusions	No data was excluded
Replication	The reproducibility of the study was confirmed by the study design which replicated the results at 3 independent facilities - University of Oxford, University of Manchester and Mary Lyon Centre, Harwell.
Randomization	Embryos were randomly allocated to experimental group on the basis of availability of plugged females.
Blinding	Because the difference in colour of the mice (albino CD1 versus agouti B6SPRTF1), the surgical procedures could not be carried out with blinding.

Reporting for specific materials, systems and methods

We require information from authors about some types of materials, experimental systems and methods used in many studies. Here, indicate whether each material, system or method listed is relevant to your study. If you are not sure if a list item applies to your research, read the appropriate section before selecting a response.

Materials & experimental systems

n/a	Involved in the study	
☒	☐	Antibodies
☒	☐	Eukaryotic cell lines
☒	☐	Palaeontology and archaeology
☐	☒	Animals and other organisms
☒	☐	Clinical data
☒	☐	Dual use research of concern

Methods

n/a	Involved in the study	
☒	☐	ChIP-seq
☒	☐	Flow cytometry
☒	☐	MRI-based neuroimaging

Animals and other research organisms

Policy information about studies involving animals; ARRIVE guidelines recommended for reporting animal research, and Sex and Gender in Research

Laboratory animals	Mus musculus (C57BL/6J) and Mus spretus between 10 and 30 weeks of age
Wild animals	The study did not involve wild-animals
Reporting on sex	The study reports on the behaviour of male fertility and as such, only cohorts of male mice were used as experimental cohorts
Field-collected samples	The study did not involve field-collected samples
Ethics oversight	Ethical approval was obtained by the Institutional Animal Welfare and Ethical Review Bodies at University of Oxford, University of Manchester and Mary Lyon Centre, Harwell.

Note that full information on the approval of the study protocol must also be provided in the manuscript.
